# First evidence of industrial fly-ash in an Antarctic ice core

**DOI:** 10.1038/s41598-023-33849-x

**Published:** 2023-04-21

**Authors:** E. R. Thomas, D. R. Tetzner, S. L. Roberts, S. D. Turner, N. L. Rose

**Affiliations:** 1 Ice Dynamics and Paleoclimate, British Antarctic Survey, High Cross, Madingley Road, Cambridge, CB3 0ET UK; 2 Environmental Change Research Centre, Department of Geography, University College London, Gower Street, London, WC1E 6BT UK

**Keywords:** Geochemistry, Atmospheric chemistry, Environmental sciences

## Abstract

Spheroidal carbonaceous particles (SCPs) are a component of fly-ash, the particulate by-product of industrial high temperature combustion of fuel-oil and coal-series fuels. We provide the first evidence that these indelible markers of industrialisation have been deposited in Antarctic ice, thousands of kilometres from any potential source. The earliest observed particle was deposited in an ice layer from 1936 CE. While depositional fluxes are low, chemical analysis of individual SCPs indicates a coal combustion origin.

## Introduction

SCPs have no natural sources, and unlike other combustion-derived components of black carbon are not produced by any other anthropogenic sources. They are primarily composed of elemental carbon making them resistant to chemical attack and are morphologically distinct under light and scanning electron microscopy^1^. These properties allow SCPs to be extracted from, and hence identified within, natural archives such as lake and marine sediments and peat sequences. Therefore, they are an unambiguous environmental indicator of industrialisation. The global scale of SCP emission provides a clear stratigraphic signal, representing a primary driving force (fossil-fuel combustion) of global anthropogenic change. Consequently, it has been suggested that SCPs may provide a useful marker for a mid-twentieth century Global boundary Stratigraphic Section and Point (GSSP) for the proposed Anthropocene Epoch. Stratigraphic records of SCP concentrations and fluxes show a major increase in response to the upsurge in demand for electricity following the Second World War, met by elevated coal combustion and the introduction of oil-fired power stations^2^.

Most historical records of SCPs within radiometrically dated sequences have been produced from lake sediments and these have been reported from every continent on Earth. Dated SCP records from marine sediments and peats have also been published but are much scarcer^3^. In the Arctic, SCPs have been recorded in cryoconite holes on the Russell glacier in southwest Greenland^4^, and in a single, coarse-resolution (7 years) ice core from Lomonosovfonna ice cap, Svalbard^5,6^. In the Southern Ocean, SCPs were detected in sediments from coastal and sub-Antarctic lakes^7^ and from near-shore marine sediments in Admiralty Bay, King George Island, Antarctica^8^. However, to our knowledge, SCPs have never been observed from an Antarctic ice core.

**Figure 1:**
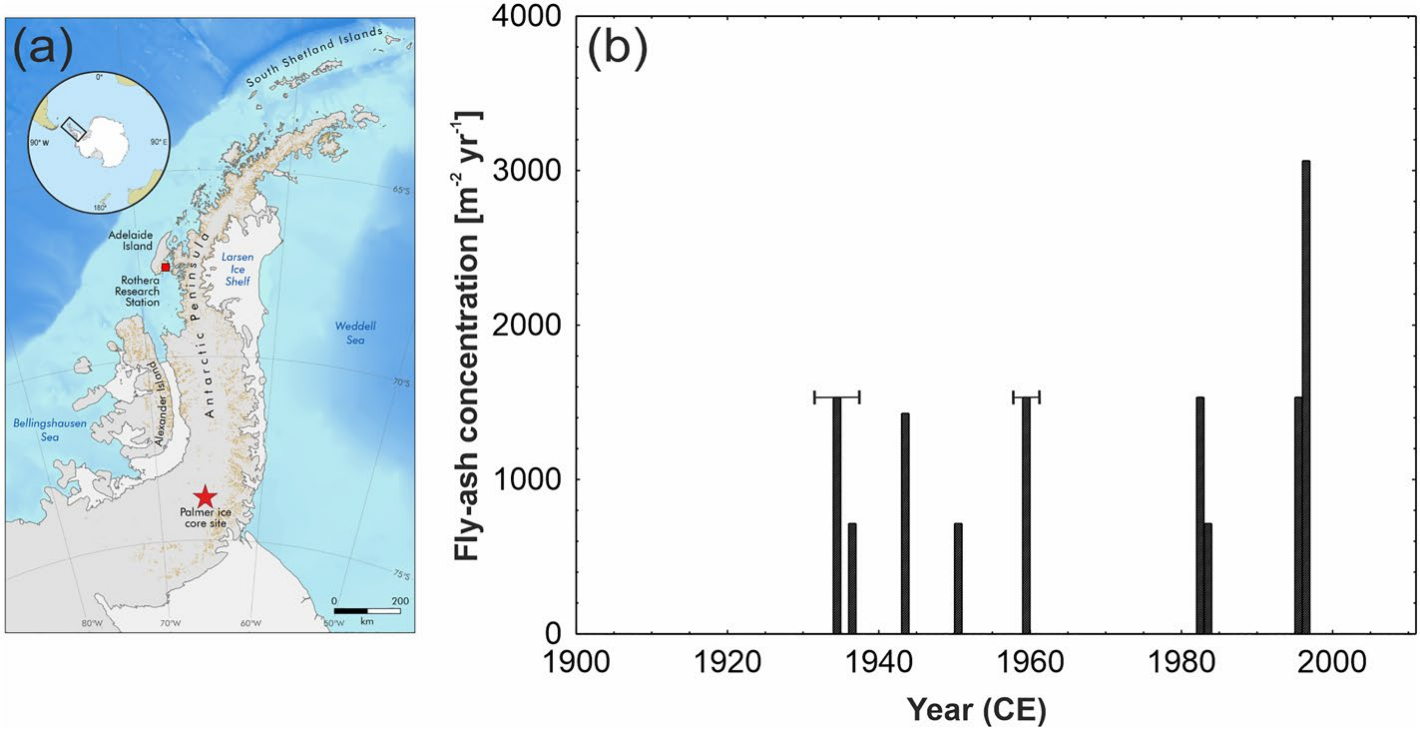
SCP deposition in the Palmer ice core. (**a**) Map of the Antarctic Peninsula, with insert of Southern Hemisphere, showing the location of the Palmer ice core (red star). (**b**) Fly-ash concentration (m^−2^ year^−1^) (1900–2011 CE) with age uncertainty shown by error bars. Map produced using the Antarctic Digital Database, using data made available under the Creative Commons Attribution 4.0 International (CC BY 4.0) licence.

In this article, we present SCP data from annually resolved samples between 1900 and 2011 taken from an ice core collected from the Palmer Land region of the Antarctic Peninsula (73.86° S, 65.46° W, 1897 m a.s.l) (Fig. 1a). SCPs were analysed as part of the work of the Anthropocene Working Group, which aims to propose a GSSP to mark the start of mid-twentieth century Anthropocene Epoch. Antarctica’s remote location, distant from sources of heavy industry and permanent human populations, may seem an unlikely location to capture a particulate marker of the Anthropocene epoch. However, here we provide the first evidence that SCPs have not only been transported to continental Antarctica, but that these enduring physical markers of fossil fuel combustion have been trapped in ice layers since the early decades of the twentieth century.

## Results

We used two independent techniques for SCP analysis, (1) chemical dissolution and light microscope detection and (2) continuous filtering and scanning electron microscope detection. The oldest potential evidence of SCPs at Palmer is found in the sample corresponding to 1930–1937 CE (Fig. 1b). However, the first definitive year, identified from the discrete sampling method at annual resolution is observed in 1936 CE.

While SCPs have been observed at many sites across the globe, few sites are more than a few thousand kilometres from the nearest industrial combustion source^2^. Even the record from the Lomonosovfonna ice core is within ~ 3000 km of several northern European sources including coal-fired power-stations on the Svalbard archipelago itself^5^. However, the closest potential contemporary sources for the Palmer ice core are ~ 4500 km away in central Chile or more than 8000 km away in southern Australia. Analysis of 5-day back-trajectories (1979–2010 CE)^9^, demonstrate that most air-masses reaching the Palmer site originate from the Bellingshausen Sea, with a smaller contribution from the Weddell Sea and the Antarctic continent. Forward-trajectory modelling^10^, from the Southern Hemisphere landmasses, suggest that South America is the dominant source region of 5-day trajectories reaching the Antarctic Peninsula. However, less frequent incursions from New Zealand and Australia are also observed. There is no evidence of air-masses from South Africa reaching the Antarctic Peninsula, at least not within the 5-day window^10^. This is supported by dust provenance studies which suggest that South America is the largest dust contributor to the Antarctic continent^11^. While the Antarctic Peninsula receives a mixture of dust from Australia and South America^12^, there is no evidence of dust derived from southern Africa.

**Figure 2:**
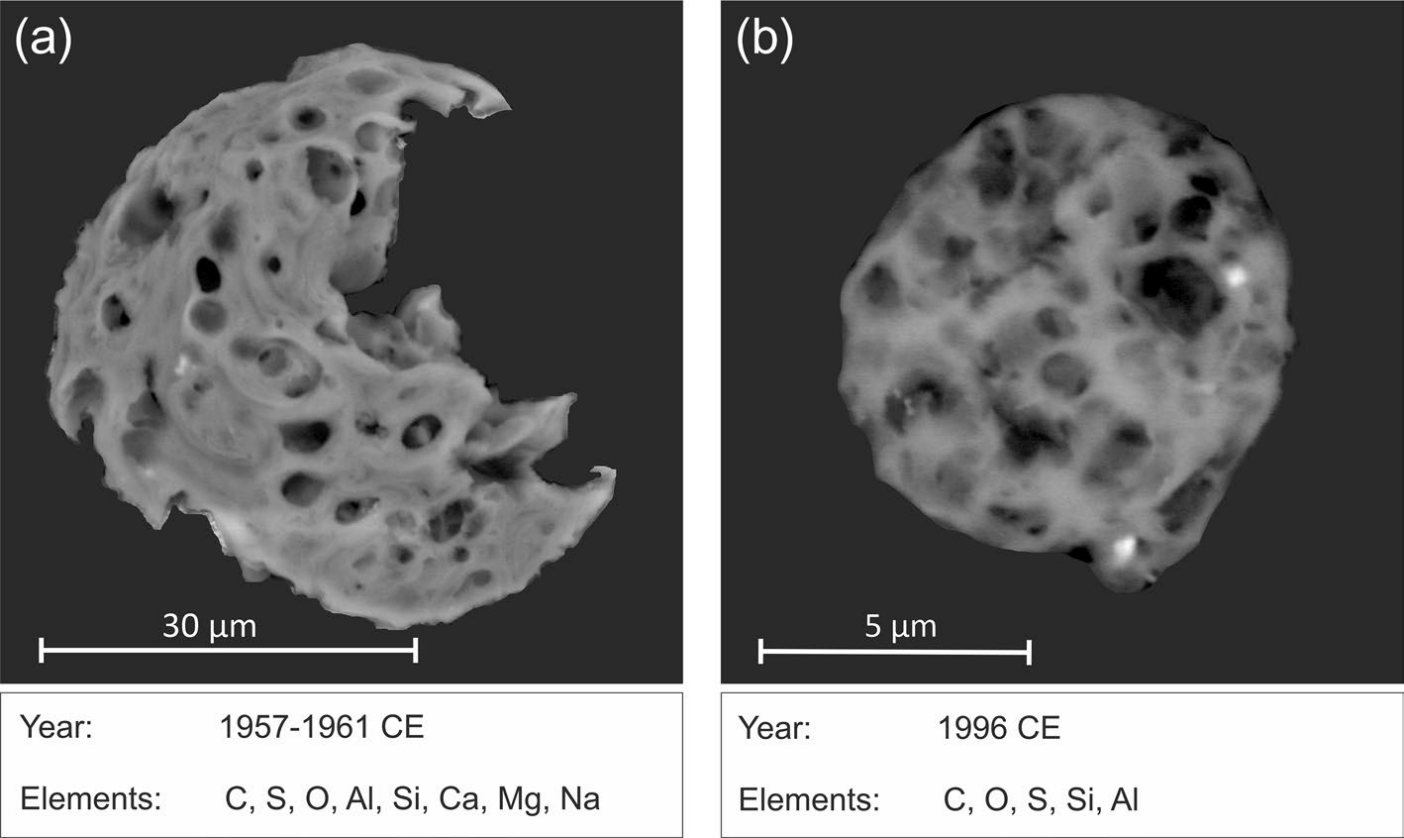
Scanning electron microscope (SEM) images. Two SCPs from the Palmer ice core showing the year of deposition and elemental composition determined by energy dispersive X-ray spectroscopy (EDS). Elements presented in order of relative intensity. SEM images presented with background filter removed.

The SCPs identified within the ice core were analysed by SEM/EDS to determine their surface chemistry and hence fuel source^13^. Three points were analysed for each SCP. In addition to carbon and oxygen, the main peaks for all SCPs analysed were found to be Al, Si and S with less frequent peaks of P, Mg, Ca and K (Fig. 2). These surficial elements on the ice core SCPs are indicative of coal combustion^13^ and are the same as those shown to be associated with coal-derived SCPs taken from a series of reference coal-fired power plants and analysed by SEM/EDS^14,15^. Importantly, this elemental analysis allows distinction from other fuel-types such as fuel-oil where Ni and V frequently occur^13,15,16^. We have not seen the elements associated with oil-fired combustion on the ice-core SCPs.

## Discussion

While their presence here is clear, the peak SCP fluxes recorded in the Palmer ice core of 3060 m^−2^ year^−1^ are amongst the lowest recorded in the world and are a factor of five lower than peak SCP fluxes in the sediments of Heart Lake in the Larsemann Hills, East Antarctica, the only other SCP data for the Antarctica mainland^6^. They are also an order of magnitude lower than peak fluxes in lake sediments from Signy Island located to the north of the Antarctic Peninsula, 13–40 times lower than in lake sediments from the Falkland Islands^6^ and a similar factor (~ 40) lower than peak fluxes reported for the Lomosovfonna ice core from Svalbard^4^. Despite evidence of increased coal-fired plants being commissioned in the Southern Hemisphere during the mid-twentieth century, we do not observe the characteristic acceleration in SCPs from the 1950s onwards as observed at other locations^2^. Indeed, there are no SCPs observed between 1960–1981 CE and this absence may indicate the importance of atmospheric circulation in fly-ash transport and deposition to the core site. The dominant mode of variability in the Southern Hemisphere is the Southern Annular Mode (SAM), which reflects the zonal pressure difference between the mid- and high latitudes. SAM governs the strength and position of the Southern Hemisphere westerly winds. During the 1960s the SAM was in its negative phase, identified as a period of weaker westerly winds, that were closer to the equator^17^. Thus, the absence of SCPs during this period may reflect a weakening in the transport regime, or a northward shift of the winds, rather than a change in the source emissions. From the 1980s onward, the westerly winds increased in strength and shifted closer to the Antarctic continent^18^. This mechanism has been attributed to the observed increase in snow accumulation in the Antarctica Peninsula^19^ and may explain the re-appearance of SCPs since 1980 and the apparent increase in 1997 CE, a period of documented strong westerly winds associated with the onset of the 1997/1998 El Niño^20^. By contrast, we attribute the absence of SCPs in the ice core prior to 1936 CE to lower levels of SCP emission and deposition combined with current limitations of analytical detection.

Here we provide the first, accurately dated, evidence of fly-ash deposition in an Antarctic ice core. The low sample concentrations, due to the small sample size, prevent a definitive attribution to a source location and we propose that this could be better addressed in a further study based on multiple Antarctic ice core locations and larger sample volumes. Our data indicate that the SCPs we have identified throughout the ice core are derived from coal combustion. Therefore, we hypothesise that the most probable source of the SCPs is Australian coal-fired power plants, with potential additional input from South America. Atmospheric circulation and transport processes have probably influenced the deposition of SCPs in Antarctica throughout the twentieth century, especially related to the strength and position of the Southern Hemisphere westerly winds. The westerly winds are predicted to increase in strength during the twenty-first century^21^, and thus we might expect to see increased deposition of fly-ash particles (and, by implication, other anthropogenically-derived atmospheric pollutants) in Antarctica in the future.

## Methods

The ice core was drilled in December 2012 (1897 m above sea level), using an electromechanical dry drill to a depth of 133.45 m. The cores were cut to 85 cm length and wrapped in layflat tubing. The ice core was dated using annual layer counting^22^, based on the seasonal deposition of non-sea-salt sulphate (nssSO_4_^2−^), hydrogen peroxide (H_2_O_2_), water-stable isotopes (δ^18^O) and methane sulfonic acid (MSA)^23^. The full core covers the period 1621–2012 CE. The estimated error in annual layer counting during the twentieth century (the focus period of this study) is less than 1-year.

The SCPs were analysed using two independent methods. The first method was based on discrete samples cut to annual resolution between 1900 to 1998 CE. The outer 5 mm of the ice core was removed prior to sub-sampling, to avoid any potential contamination from field handling, transport, or storage. The frozen samples were melted in sealed sterile low-density polyethylene (LDPE; Nalgene™) bottles and filtered through Whatman™ glass microfibre filters. Due to the varying snow accumulation at Palmer, the annual liquid sample volumes ranged from 64 to 430 mL per year. SCPs are composed mostly of elemental carbon and are chemically robust. Thus, other particulate material and the filter paper were removed, using a series of acid treatments including hydrofluoric acid followed by hydrochloric acid^14^. Between acid treatments, the samples were washed with distilled water, centrifuged, and decanted to remove any remaining acid. The final suspension was evaporated onto multiple coverslips and mounted onto microscope slides where the number of SCPs was counted using a light microscope at × 400 magnification using the criteria described in^1^. The SCP count was converted to a flux, based on the known ice core sample area of 11.4 cm^2^. All digestion apparatus was rigorously cleaned prior to use and filtration equipment was washed with deionised water in between each sample. All digestion steps from filtration and digestion through to slide preparation were undertaken in a laboratory clean room, inside a fume extraction cupboard. In between stages, all samples were kept covered even within the fume extraction cupboard.

The second method sampled SCPs from the meltwater collected during the continuous flow analysis (CFA). The CFA section is taken from the central part of the ice core to avoid contamination. Melt water was collected in sealed low-density polyethylene (LDPE; Nalgene™) bottles, sampled at approximately annual resolution during the period between 1980–2011 CE. Samples corresponding to years 1900–1979 CE were sampled at approximately five to eight-year resolution (16 samples). Sample volumes ranged between 44 and 927 mL. The meltwater was filtered through 13 mm diameter, 1.0 μm pore size Whatman™ polycarbonate membrane filters, inside clean polypropylene Swinnnex™ filter holders. Each filter was then mounted onto an aluminium stub for analyses on a Quanta-650F Scanning Electron Microscope (SEM) using back scattered electrons (BSE) on a low-vacuum mode. Each filter was imaged at × 800 magnification for SCPs identification^24^. The conversion to concentration was based on an estimated ice core surface volume through the melt-head of 6.53 cm^2^. In both methods, analytical blanks were included during the melting and filtration process. No SCPs were observed in the blanks of either method.

SCP surface chemistry analyses were conducted using a Quanta-650F Scanning Electron Microscope (SEM) equipped with two Bruker XFlash 6130 detectors for energy dispersive X-ray spectroscopy (EDS). The SEM/EDS setup was configured to work at 15 kV on a low-vacuum mode. Three spot analyses were performed on each SCP.

## Data Availability

The data generated as part of this study is available at the UK Polar Data Centre https://www.bas.ac.uk/data/uk-pdc/). The age-scale is available at 10.3390/geosciences12020087 and the SCP count and SEM images presented in this study are available from the corresponding author on request.
